# Single-cell assessment of iron content in primary human T cells using laser ablation inductively coupled plasma mass spectrometry

**DOI:** 10.1016/j.crmeth.2026.101343

**Published:** 2026-03-26

**Authors:** Diana M. Carp, Piotr Golda, Alexander Griffiths, Katie Flaherty, Alexander Morrell, Anna Schurich

**Affiliations:** 1 Department of Infectious Diseases, School of Immunology and Microbial Sciences, King’s College London, London SE1 9RT, UK; 2 London Metallomics Facility, Faculty of Life Sciences & Medicine, King’s College London, London, UK; 3 Department of Earth Sciences and Engineering, Imperial College London, London, UK

**Keywords:** single-cell metallomics, iron quantification, LA-ICP-MS, ICP-MS, T cells, metalloimmunology, iron metabolism, single-cell analysis, human donors

## Abstract

Transition metals, such as iron, support vital metabolic and signaling functions in immune cells. Cellular iron concentrations are tightly controlled. In T cells, both iron deficiency and iron overload have been linked to immune dysfunction. Homeostatic iron concentrations in T cells, and changes that occur during T cell activation, remain poorly understood due to difficulty of accurately measuring iron content in single cells, especially in small cells. Here, we describe the use of laser ablation inductively coupled plasma mass spectrometry (LA-ICP-MS) to accurately quantify the total amount of endogenous iron in individual primary human T cells. Our technique allows for targeted selection of single cells and reproducible quantification of iron at femtogram level. Our findings reveal that iron levels in resting T cells were similar across human donors. In contrast, T cell activation leads to diverse patterns between individual cells and donors, indicating specialized needs during differentiation.

## Highlights

• Single-cell analysis of metal content by laser ablation coupled mass spectrometry
• Quantification of iron in small primary suspension cells using LA-ICP-MS
• Suitability of method for assessment of fixed and unfixed cell samples
• Resting human primary T cells contain on average 1.38–2.23 fg iron per cell

## Motivation

Transition metals, such as iron, play important roles in cellular function. However, the accurate quantification of metal content on a single-cell level has been challenging. Here, we introduce a method making use of laser ablation inductively coupled plasma mass spectrometry to sensitively quantify iron levels in primary human T cells. Our method will enable future detailed studies into the biological relevance of metals for cellular function.

## Graphical abstract

**Figure:**
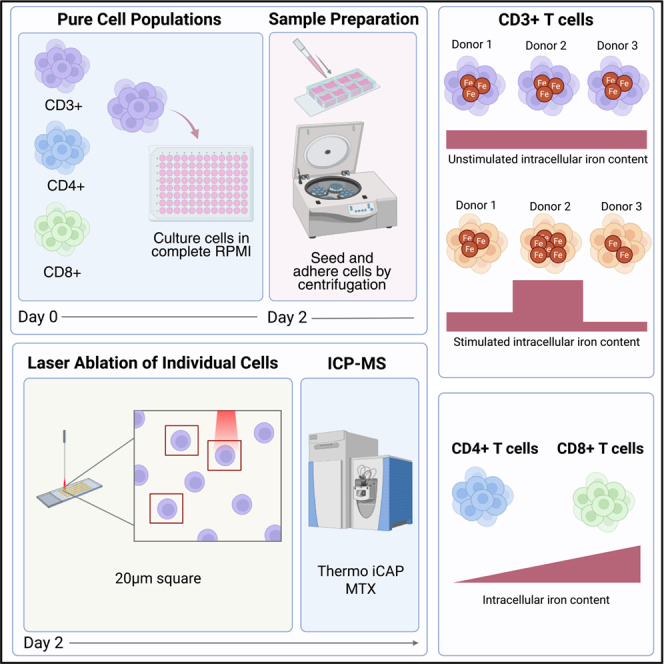

## Teaser

Iron is an essential transition metal necessary for metabolic and signaling functions in immune cells. Carp et al. present a method for the preparation of human T cells for visual inspection and analysis of intracellular iron content at a single-cell level using laser ablation inductively coupled mass spectrometry (LA-ICP-MS).

## Introduction

Immune cells have highly specialized metabolic demands to fuel and regulate their functions.^1^^,^^2^ Naïve and resting T cells have a low metabolic and proliferative rate, however, upon activation T cells reprogram their metabolism to meet the increased energetic cost of proliferation and effector molecule production.^1^^,^^3^ Transition metals play an important role in T cell immunity, for example, in the regulation of signaling events and as part of co-factors in metalloproteins.^4^^,^^5^ Iron is a key trace metal necessary for a wide range of biological process, from DNA synthesis, energy metabolism, and oxygen transport, to forming catalytic domains in enzymes as part of iron sulfur clusters.^6^^,^^7^ Upregulation of the iron transporter CD71 (transferrin receptor 1) is a phenotypic marker of T cell activation in line with increased cellular iron requirements during effector differentiation.^8^ Consequently, iron deficiency negatively impacts T cell immunity,^5^^,^^9^^,^^10^ and ablation of functional CD71 has been shown to impair T cell proliferation and results in immune-deficiency.^11^^,^^12^ In contrast, a high cellular iron load has been observed in macrophages in chronic obstructive pulmonary disease^13^^,^^14^ and in T cells in Lupus erythematosus.^15^^,^^16^ While these findings underline the importance of iron regulation for healthy immunity, the precise iron content of individual T cells and the resultant relation to specific phenotypic and functional characteristics remain poorly understood. Using colorimetric assays and histological staining methods on bulk cell populations^5^^,^^17^ or fluorescent probes for single-cell analyses has provided qualitative, but not specific quantitative insight.^13^^,^^18^ The ability to accurately quantify the iron content within T cells would enable investigation into the role of iron metabolism.

Inductively coupled mass spectrometry (ICP-MS) remains the gold standard for sensitively quantifying metals in biological specimens.^19^ The use of single cell (sc)-ICP-MS, which utilizes a micro-fluidics flow-based system to quantify transition metals in cell suspension has been demonstrated for large cells, like macrophages and metallodrug-treated cancer cells, as well as those with naturally high metal content, such as red blood cells.^19^^,^^20^^,^^21^^,^^22^^,^^23^ While sc-ICP-MS enables high throughput sample introduction, it requires the cells to be fixed and needs careful fluidic control to ensure single cells are introduced without contamination by cell aggregates or debris.^19^

Here, we introduce an alternative method for sample introduction by coupling a laser ablation system to inductively coupled plasma mass spectrometry (LA-ICP-MS) and enable measurement of iron content in single primary human T cells.

LA-ICP-MS provided us with several advantages. Briefly, single-cell LA-ICP-MS involves the process of individually selecting a cell for ablation, using the laser to remove them sequentially from a glass slide. The cells are introduced quickly into an ICP-MS where the constituent elements within the cell are atomized, ionized, filtered using quadrupoles and finally detected. The microscope-camera system within the laser ablation unit allows for visual inspection and selection of intact single cells to be analyzed, ensuring exclusion of cell aggregates and debris. This technology is suspension-free assessment, limiting background signal noise and potential contamination from the suspension fluid. Post hoc analysis and ambiguity over the potential quantification of cell doublets, cell debris, or contamination from sheath fluid is thereby minimized. Finally, we find that LA-ICP-MS requires minimal sample preparation making it compatible with assessment of fixed and unfixed cells; enabling assessment where cells cannot be fixed and/or further avoiding alterations to cellular metal content introduced by the fixation process.

Our data reveals that iron content in resting T cells across healthy donors is highly similar. In contrast, upon activation T cells show striking donor-specific variation. Taken together, we here demonstrate the use of LA-ICP-MS to sensitively quantify the endogenous iron content in individual primary T cells. Our method will enable future detailed studies into the biological role of iron in cellular metabolism and function and might reveal novel targets for therapeutic intervention.

## Results

### LA-ICP-MS provides the required sensitivity to assess endogenous iron content in individual primary T cells

We set out to quantify intracellular iron with high sensitivity in individual cells. To ensure pure T cell populations for single-cell analysis, we used fluorescence-activated cell sorting (FACS) to sort CD3^+^ T cells from peripheral blood mononuclear cells (PBMC) from healthy donors, with no known iron deficiency (*donor information in* Table S1). To mitigate against metabolic stress induced by cell sorting,^24^^,^^25^ T cells were then rested in culture for 40 h in iron sufficient media (RPMI supplemented with 10% FCS and 20 IU IL-2) prior to LA-ICP-MS analysis.

**Figure 1:**
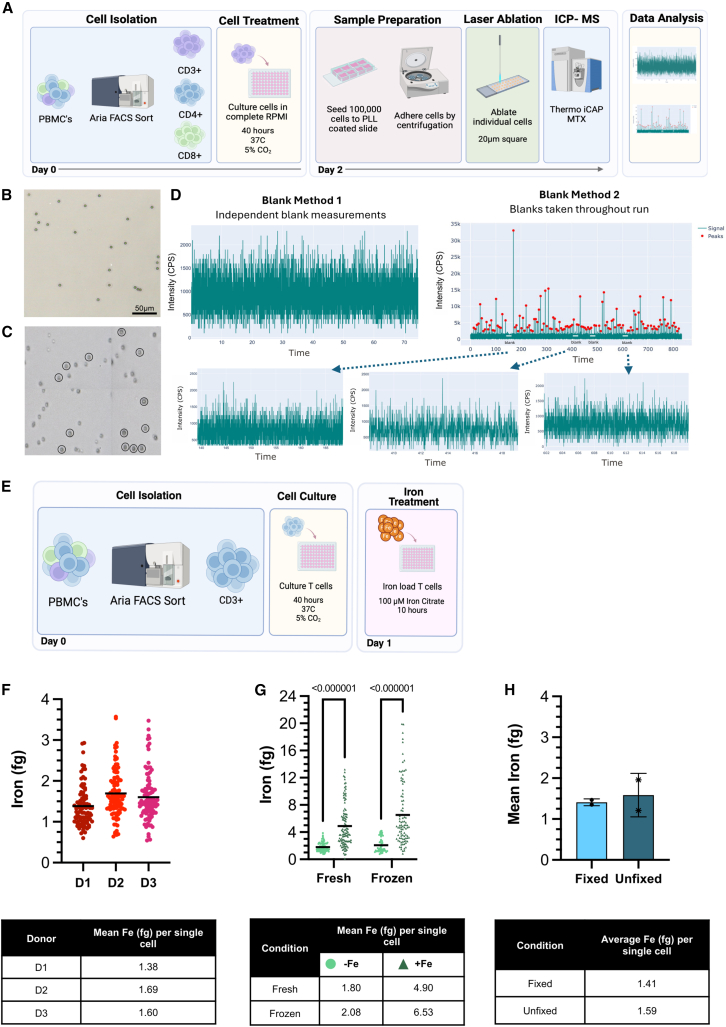
LA-ICP-MS provides the required sensitivity to assess endogenous iron content in individual primary T cells (A) Experimental set up, briefly, flow-sorted CD3^+^ T cells were counted and plated in MS buffer onto poly-L-lysine-coated removable 8-well chambers. Cells were gently centrifuged to promote adherence. All remaining buffer was removed by aspiration, and cells were subsequently washed by metal trace water. Cells were visually selected for laser ablation and iron content assessed by ICP-MS. (B and C) Visualization of adherent T cells by light microscopy (upper image) and on LA-ICP-MS connected system (lower image) selected cells for ablation are marked. (D) Complementary techniques for obtaining blank measurements from the slide in areas without cells. Blank method 1 (left), obtaining blank measurements independently of other measurements. Blank method 2 (right), involves taking blank measurements throughout the ICP-MS run and confirms the slide is free external analyte contamination. Highlighted throughout the run are blank measurements obtained throughout one continuous ICP-MS analysis run. (E) Experimental workflow for iron loading. Cells were FACS sorted into pure CD3^+^ T cell populations and cultured for 40 h, with 100 μM of iron citrate added in the last 10 h of culture prior to LA-ICP-MS analysis. (F) Intracellular iron (fg Fe) per CD3^+^ sorted T cells for 3 donors, with table showing mean Fe per single cell (horizontal line). Each dot represents iron content per single cell. nD1 = 89, nD2 = 104, nD3 = 99. (G) Single-cell iron quantification for fresh and frozen unstimulated CD3-sorted T cells without and with iron loading (100 μM). The bar represents mean fg of Fe per single cell. Statistical significance was determined using a multiple Mann-Whitney test; *p* value written above. Each symbol represents a single cell from D8; n fresh,-Fe = 91, n fresh,+Fe = 112, n frozen,-Fe = 49, n frozen,+Fe = 99. (H) Pooled data from two donors (D1 and D13) of single-cell iron quantification (fg) of fixed (mean fg per cell = 1.41) and unfixed (mean fg per cell = 1.59). Each symbol represents the mean intracellular iron from an individual donor; *n* = 2 donors.

Analysis by LA-ICP-MS requires cells to be adherent, we therefore developed a method for gently adhering T cells to the assay glass slides with limited cell fragmentation. Briefly, T cells were harvested from culture, washed, and taken up into our MS solution, containing filtered HEPES with 2 mM EDTA and 2% PFA fixative. T cells were then distributed evenly spaced out across a poly-L-lysine-coated glass chamber slide. To ensure complete adherence, cells were centrifuged onto the slides for 4 min at 470 g (Figure 1A). MS solution was removed completely by aspiration, and the slides were gently washed with type 1-ultra pure water prior to laser ablation. Slides were then visually inspected to ensure there was no undo cell damage, and cells appeared intact (Figure 1B). This preparation for LA-ICP-MS allows subsequent selection of individual cells for laser-ablation. T cells are relatively small cells with an average diameter of 5–12 μm. A spot size of 20 μm was selected to ensure single T cells were completely captured. For each cell ablation, intensity was measured and recoded in counts per second (CPS), during a timed interval (Figures 1C and 1D). Gel microdroplets with known analyte concentrations were also ablated to acquire a calibration curve and determine iron content at femtogram sensitivity (Figure S1). To control for potential contamination from any residual culture media or MS solution, we collected blank measurements from multiple areas on the analyzed chamber slide to serve as “blank measurements” for the CPS background threshold. We tested two methods of collecting blank measurements, either taken independently (before or after all cells were acquired) or throughout the run collecting blanks after every 20 cells assessed (Figure 1D). We found no difference in the CPS intensity between the two methods. For ease of experimentation, we opted to collect blank measurements independently.

For all runs (*experimental set up in* Figure 1A), between 70 and 120 cells were recorded per sample and analyzed based on the LA-ICP-MS parameters described in the STAR Methods section. Despite the small size and thus cellular material available per cell, we were able to quantify iron on a single-cell level with high sensitivity (femtogram, 10^−15^ g), detecting a mean iron content ranging from 1.34 to 1.69 fg in rested T cells derived from fresh (not previously cryopreserved) samples across 3 individual donors (Figure 1F). Iron can be particularly challenging to measure at low quantities in biological samples using ICP-MS due to the polyatomic interferences of ArO and CaO. These molecules possess the same mass as the most abundant isotope (56Fe). Therefore, it was a necessity to use a triple-quadrupole-based instrument with a collision reaction cell with O_2_ to remove these interferences to allow high sensitivity and selectivity of iron. To further validate the specific detection of iron, we next iron loaded T cells (Figure 1E) in culture using iron (III) citrate at 100 μM concentration. Iron loading increased the cellular iron content by 2- to 3-fold (Figure 1G). We also compared the use of T cells isolated from fresh or cryopreserved PBMCs. Differences in T cell metabolism have been observed after cryopreservation, with a decrease in T cell expansion, activation, and increased mitochondrial oxidative stress.^26^^,^^27^^,^^28^ We find that after the 40 h culture period, previously cryopreserved T cells show no deficit in iron uptake compared to cells isolated from fresh PBMC from the same donor (*p* = 0.508) (Figure 1G). Fluidics-based systems currently do not allow to assess unfixed cells; however, fixation of cells could lead to changes of intracellular metal ion levels. In tissue samples, leaking has been recorded for some transition metals, while for iron there was insignificant detectable change reported.^29^^,^^30^ To investigate whether fixation potentially impacted iron levels in T cells, we tested if LA-ICP-MS could be used to assess unfixed cells. We used the same protocol as before, except that washes with ultrapure water were replaced by HBSS. Unfixed cells could indeed be assessed, and we found comparable intracellular iron content in fixed and unfixed cells in two donors, 1.41 vs. 1.59 fg, respectively (Figure 1H). Thus, LA-ICP-MS enables analysis of iron in both fixed and unfixed cells; however, we opted for using fixed cells for all subsequent experiments.

### Accuracy of LA-ICP-MS measurements as validated by bulk ICP-MS

**Figure 2:**
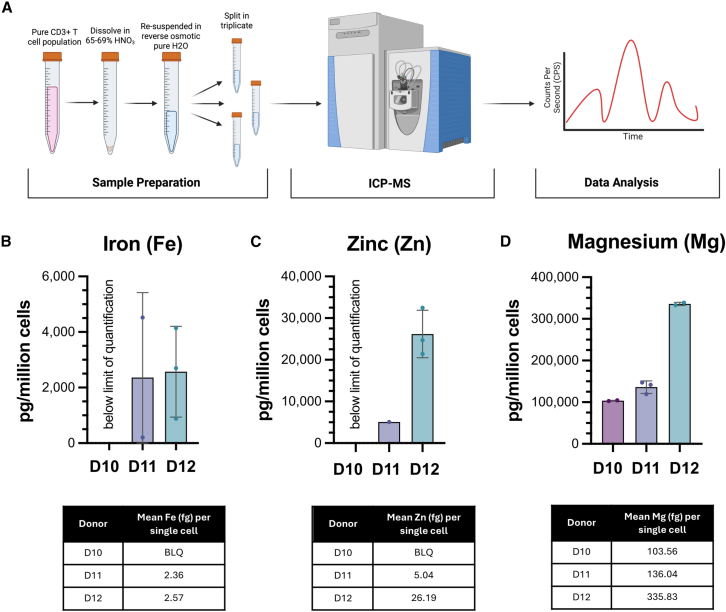
Accuracy of LA-ICP-MS measurements as validated by bulk ICP-MS (A) Schematic of the workflow analysis for bulk ICP-MS. T cells were counted and digested in 65%–69% HNO_3_ before being resuspended in reverse osmotic pure H_2_O and split into triplicates. Samples were run on the NexION5000. (B–D) Metal quantification of iron (Fe), zinc (Zn), and magnesium (Mg) quantified in pg/million cells in activated CD3 T cells for three healthy donors (*n* = 3). Tables below represent mean fg/cell converted from pg/million cells for each donor. BLQ, below the limit of quantification.

Next, we validated our single-cell measurements against an established method of bulk-cell mass spectrometry, an approach previously used to quantify the concentration of iron in lymphocytes and macrophages.^31^ To ensure sufficient T cell numbers for analysis, we first expanded T cells *in vitro*. Briefly, PBMCs from three individual donors were activated by plate bound anti-CD3/CD28 and expanded for 9 days in culture before cells were FACS sorted and rested for bulk analysis. Four million pure T cells per donor were digested with HNO_3_ and resuspended in reverse osmotic deionized water in preparation for bulk population ICP-MS analysis on a NexION5000 (Figure 2A). Cells were split for the analysis of technical triplicates. We analyzed the following elements: iron (Fe), zinc (Zn), and magnesium (Mg) (Figures 2B–2D). After converting iron content from bulk ICP-MS to femtogram per single cell, we obtain 2.36 and 2.57 fg, respectively, for two donors resembling the measurements obtained with LA-ICP-MS (Figure 2B).

Additionally, we found 5.04 and 26.19 fg zinc per cell (Figure 2C). For both iron and zinc, elements found at endogenously low levels, we observed high variation between the technical triplicate sample repeats, and we obtained no quantifiable measurement for one of the three donors (Figures 2B and 2C). These small margins for error had negligible impact on assessing magnesium, an element present at higher concentrations (Figure 2D). Values for iron content calculated from bulk quantification for single cells were slightly higher than we had observed in our direct single-cell LA-ICP-MS analysis. A possible reason for this could be that cells for bulk analysis had been previously activated to expand numbers *in vitro*. Since T cells increase metabolic activity upon activation, these likely impacts iron uptake. Using single-cell LA-ICP-MS allows for a 40-fold reduction in cells required for an assay, down from 4 million to 100 thousand cells enabling acquisition of rare cell populations such as from clinical samples.

### LA-ICP-MS analysis identifies difference in iron content between T cell subsets

**Figure 3:**
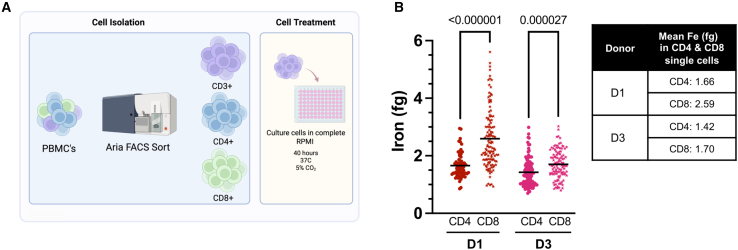
LA-ICP-MS analysis identifies difference in iron content between T cell subsets (A) Representative figure of experimental workflow. PBMCs were FACS sorted for CD3^+^, CD4^+^, and CD8^+^ T cells and cultured for 40 h in complete RPMI. Intracellular iron was quantified by LA-ICP-MS. (B) Intracellular iron (fg Fe) per cell CD4^+^ and CD8^+^ sorted T cells for D1 and D3 (shown in Figure 1F). Statistical significance was determined using a multiple Mann-Whitney test; *p* value written above. Each symbol represents a single cell, nD1, CD4 = 63, nD1, CD8 = 122, nD3, CD4 = 104, and nD3, CD8 = 118, and mean values are represented by black horizontal line.

Previous studies on CD4 and CD8 T cells have revealed metabolic differences^32^^,^^33^ and work from Teh et al. predicted higher iron content in murine CD8 versus CD4 T cells using mathematical modeling.^5^ To experimentally assess potential differences in T cell populations, we FACS sorted CD4 and CD8 positive lymphocytes from two donors (D1 and D3) for LA-ICP-MS analysis as before (Figure 3A). We find that CD4 T cells in both donors had a lower endogenous cellular iron content in comparison to their matched CD8 counterparts (Figure 3B) in line with the predictive model.^5^

### Single-cell analysis reveals changes in iron content upon T cell activation

**Figure 4:**
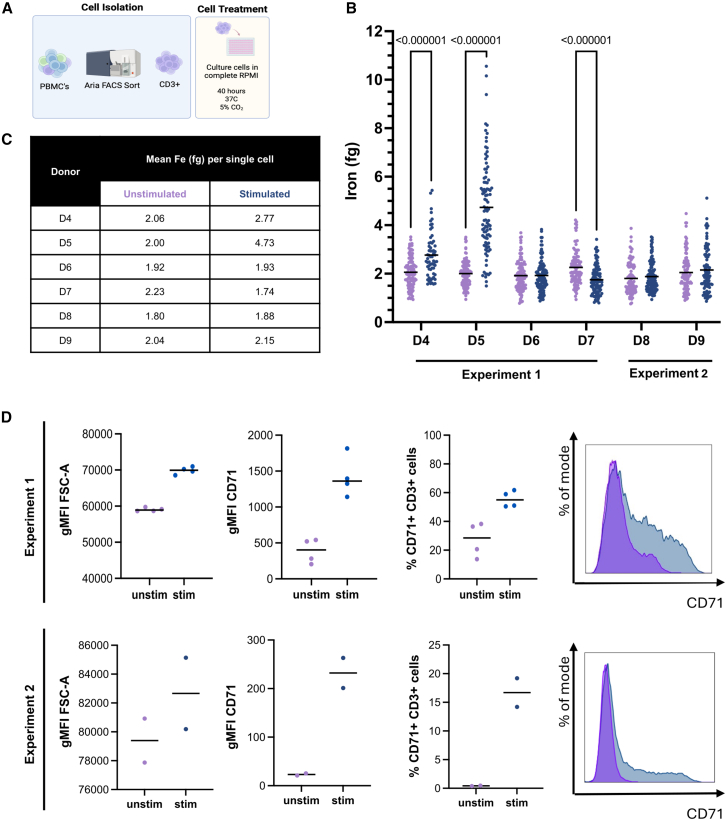
Single-cell analysis reveals changes in iron content upon T cell activation (A) Representative figure of experimental workflow. PBMCs were FACS sorted for CD3^+^ T cells and cultured for 40 h in complete RPMI with CD3/CD28 stimulation. Subsequently intracellular iron was quantified by LA-ICP-MS. (B) Intracellular iron quantification per single cell for 6 donors, for stimulated (blue) and unstimulated (purple) CD3^+^ T cells. Data are shown from two independent experiments. Statistical significance was determined using a multiple Mann-Whitney test, significant *p* values written above. Donors 4–7 and donors 8–9 were analyzed in two independent experiments. Each individual symbol represents an single cell analyzed and mean values are represented by black horizontal line; nD4,unstim = 101, nD4,stim = 62, nD5,unstim = 110, nD5,stim = 94, nD6,unstim = 107, nD6,stim = 103, nD7,unstim = 99, nD7,stim = 100, nD8,unstim = 91, nD8,stim = 114, nD8,unstim = 99, nD9,stim = 91. (C) Summery data table of mean Fe (fg) per single for stimulated and unstimulated CD3^+^ sorted T cells. (D) Relative size of stimulated and unstimulated T cells, surface expression of transferrin receptor, CD71, in stimulated (blue) and unstimulated (purple) T cells, and representative histogram of CD71 surface expression in stimulated and unstimulated T cells by flow cytometry, normalized to the mode. Shown for two independent experiments for the donors analyzed in (C), *n* = 6 donors. Mean values are represented by a horizontal black line throughout figure.

Successful activation of T cells induces proliferation and effector functions which require increased nutrient uptake.^2^^,^^34^ These are very energetically demanding processes necessitating increased nutrient uptake, including amino acids, glucose, extracellular lipids and iron. Increased expression of the iron transporter, transferrin receptor 1 (CD71), is a hallmark of T cell activation.^8^^,^^35^ We therefore investigated whether upon activation iron content in T cells would increase. FACS-sorted T cells were either left resting or were activated by generic stimulation with plate bound anti-CD3/CD28 in culture for 40 h (Figure 4A). We found highly varied responses between the 6 individual donors assessed; however, upon activation we found that intracellular iron content correlates with previously obtained bulk ICP-MS (Figure 2B). While in donors 4 and 5, particularly, T cell activation led to a strong increase in cellular iron levels (Figure 4B), comparable to those found when iron loading T cells in previous experiments (Figures 1F and 1H), in other donors we found unchanged or even mildly reduced levels of cellular iron after stimulation. This finding was unexpected, as comparable T cell activation was confirmed in all donors by flow-cytometric analysis, demonstrating cells were increasing in size (blasting) as measured by increased forward scatter area (FSC-A) (Figure 4D) and had increased iron-uptake capacity, as the frequency and per cell expression of transferrin receptor (CD71) increased (Figure 4D). Our data suggests diverse metabolic needs in activated T cells; therefore, direct measurement of iron content will be valuable in future investigations.

## Discussion

Recently, there has been increased interest in understanding the biological role of metals in cells.^36^ There is a surge of therapeutic research using sc-ICP-MS to quantify the efficacy of metallodrugs in various cancer cell line models,^37^^,^^38^ and the field of neuroscience has benefited from the use of LA-ICP-MS to study metal distribution within tissues sections in neurodegenerative and ischemic diseases, such as Parkinson’s and Alzheimer’s diseases.^39^^,^^40^^,^^41^ While an important role for transition metals in the cellular metabolism is emerging, accurate single-cell quantification remains challenging. Iron availability has been shown to play a vital role in T cell function altering key metabolic pathways, influencing epigenetic changes and ultimately impacting the immune response.^42^^,^^43^ Quantifying iron content at the single-cell level represents the initial step toward understanding the impact of iron on specific T cell traits, which could enable future therapeutic targeting.

We report the development and application of a selective and sensitive LA-ICP-MS protocol for quantifying endogenous iron at single-cell resolution in T cells. Although lower throughput than fluidics-based systems, LA-ICP-MS offers significant advantages. By immobilizing cells on glass slides and removing residual buffer, individual intact cells can be visually identified and precisely ablated, enabling targeted analysis while minimizing contamination from debris, aggregates, or extracellular iron. Notably, iron levels in fixed and unfixed cells were comparable, supporting the novel use of LA-ICP-MS for assessment of unfixed cells for direct iron quantification.

We validated our measurements against established bulk ICP-MS.^44^^,^^45^ While both methods produced comparable overall results, we found high variability and substantial technical noise in bulk-ICP-MS measurements for low abundance analytes, iron, and zinc, in our cell populations. Accurate quantification of endogenous iron would necessitate increased cell input per donor and additional technical replicates, while no constraint in the assessment of homogeneous cell-lines, it precludes the study of rare and heterogeneous primary cell populations.

Using single-cell LA-ICP-MS analysis, we found low variability in iron levels in unstimulated T cells within samples and among donors (1.83 fg iron ± 0.27), likely reflecting tight regulation of iron homeostasis and low metabolic activity of resting T cells. Post stimulation, when T cells increase their metabolic needs endogenous iron levels were distinctly donor dependent and highly variable.^1^^,^^2^^,^^46^ The underlying cause of this heterogeneity in intracellular iron levels remains to be elucidated but may reflect dynamic shifts in metabolic requirements during T cell proliferation and differentiation. In contrast, the transferrin transporter CD71 was upregulated in activated T cells from all donors. While not applicable to our *in vitro* culture conditions, CD71 expression is also induced under conditions of iron deprivation.^42^^,^^47^ These observations suggest that CD71 expression levels reflect the requirement for, rather than the present content of intracellular iron at a given point in time.^48^ Finally, when analyzing global CD3^+^ T cell samples, the frequency and activation status of CD4 vs. CD8 positive T cells within the population could skew results, as we find higher intracellular iron levels in CD8 T cells in accordance with earlier predictive models.^5^

Together, our study establishes LA-ICP-MS as a powerful method for dissecting transition metal heterogeneity in single cells. This will enable future detailed investigation of cellular metal metabolism and its relevance in organismal health and disease.

### Limitations of the study

A current limitation of our method is the time-consuming manual selection of individual cells for ablation, restricting the number of cells analyzed. Our approach could be complemented by microfluidic systems enabling higher cell throughput. Faster single-cell analysis could further be achieved by automating cell selection and improving the integration of laser and mass spectrometry technologies.

Our sample preparation has been optimized to ensure an iron-free environment; however, measuring other elements such as calcium, potassium, sodium, and phosphorus will require use of alternative specialized free buffer solutions. Intense washing in pure water could damage cellular integrity and cannot be applied during assessment of unfixed cells. In the future, we aim to couple laser ablation with inductively coupled plasma time of flight mass spectrometry. This will allow us to simultaneously quantify a comprehensive panel of transition metals and/or combine endogenous metal quantification with analysis of cellular phenotypic and functional markers. In the future, the use of elemental tagging could be explored to further investigate different cellular activation states or discern distinct T cell populations, e.g., within mixed PBMC populations, bypassing the need for FACS sorting. This will be indispensable to further understand the correlation between individual cellular characteristics and transition metal profile.

## Resource availability

### Lead contact

• Requests for further information and resources should be directed to and will be fulfilled by the lead contact, Anna Schurich (anna.schurich@kcl.ac.uk).

### Materials availability

• This study did not generate new unique reagents.

### Data and code availability

• Original data are available upon request from the lead contact, Anna Schurich.
• This paper does not report original code.
• Any additional information required to reanalyze the data reported in this paper is available from the lead contact upon request.

## Acknowledgments

This work was supported by the 10.13039/501100000268Biotechnology and Biological Sciences Research Council (BB/T008709/1) to D.C. and Medical Research Council, Molecular and Cellular Medicine Board (MRC MCMB) (MR/Z504269/1) to A.S. We acknowledge the London Metallomics Facility (https://www.kcl.ac.uk/research/facilities/london-metallomics-facility) for their support in generating and analyzing data presented within this manuscript. We also acknowledge the support from the 10.13039/501100000921European Cooperation in Science and Technology (COST) Action CA21115. Some figure elements were created using BioRender.com. Finally, we would like to kindly thank all the donors for their generous blood donation that made this research possible and the wonderful phlebotomy team for all their support and care with sample collection.

## Author contributions

Conceptualization, A.S.; methodology, A.G., A.M., and D.M.C.; investigation, D.M.C. and K.F.; visualization, D.M.C.; writing – original draft, A.S. and D.M.C.; writing – review and editing, all authors; funding acquisition, A.S.; supervision, A.S.

## Declaration of interests

The authors declare no competing interests.

## STAR★Methods

### Key resources table

**Table:** 

REAGENT or RESOURCE	SOURCE	IDENTIFIER
**Antibodies**		

CD3 anti-human (APC-Cy7, clone: SK7)	Biolegend	Cat#344818; RRID: AB_10645474
CD4 anti-human (PE Cy7, clone: OKT4)	Biolegend	Cat# 317414; RRID: AB_571959
CD8 anti-human (AF700, clone: OKT8)	Invitrogen	Cat# 56-0086-82
CD71 anti-human (Percp-Cy5.5, clone: CY1G4)	Biolegend	Cat# 334114; RRID: AB_2563175
Ultra-LEAF™ Purified anti-human CD3 Antibody (clone:OKT3)	Biolegend	Cat# 317326; RRID: AB_11150592
Ultra-LEAF™ Purified anti-human CD28 Antibody (clone: CD28.2)	Biolegend	Cat# 302934; RRID: AB_2616667

**Biological samples**		

Healthy adult blood samples		N/A

**Chemicals, peptides, and recombinant proteins**		

Iron (III) Citrate	Sigma	CAT#F6129-250G
Poly-L-Lysine	Sigma	P8920-100ML

**Software and algorithms**		

FLowJo Software v10	FlowJo, LLC	https://www.flowjo.com/
GraphPad Prism	GraphPad Software, LLC	v10.6.1
Visual Studio Code	Microsoft	v1.106.3
Ananconda	Anaconda Inc	V2.6.3

**Other**		

Transact	Miltenyi	130-128-758
RPMI 1640	Gibco	31870–025
HEPES	Sigma	H0887-100mL
Non-essential amino acids	Gibco	11140–050
Sodium- Pyruvate	Gibco	11360–070
HBSS, no calcium, no magnesium, no phenol red	Gibco	Cat# 14175053
4% Paraformaldehyde, in 1× PBS PH7.4 Solution	Severn Biotech	Cat# 40-7401-05
Lymphopure	Biolegend	Cat# 426202
Nunc™ Lab-Tek™ II Chamber Slide 8-well Chamber Slide w/removable wells	Thermo Scientifc	Cat# 154534PK
Purified Metal Grade Water (resistivity ≥18.2 MΩ)	Merck	Milli-Q IQ 7015
Quadrupole inductively coupled plasma mass spectrometer (for single cell analyses)	Thermo Fisher Scientific	iCAP MTX
Laser Ablation system	Teledyne Photon Machines	Iridia 193 nM
Quadrupole inductively coupled plasma mass spectrometer (for bulk analyses)	PerkinElmer	NexION5000
Microgelatin droplets	University of Vienna	BIO-logi-CAL
Standard glass reference material	National Institute for Standards and Technology (NIST)	(SRM) 612 glass

### Experimental model and study participant details

#### Ethics statement

Blood samples were obtained from healthy donor volunteers under the Research Ethics Committee permission (HR/DP-21/22–14568). All subjects gave their written informed consent. The study was conducted in accordance with the Declaration of Helsinki. All storage of samples obtained complied with the requirements of the Data Protection Act 1998 and the Human Tissue Act 2004, issued by the UK parliament. Study participant details can be found in Table S1.

### Method details

#### T cell culture for bulk analysis

Peripheral blood mononuclear cells (PBMCs) were isolated from whole blood by density gradient centrifugation (Lymphoprep, STEMCELL Technologies, Cambridge, UK). Subsequently, cells were cultured in complete RPMI 1640 medium (cRPMI) supplemented with 2mM L-glutamine, 0.1mM non-essential amino acids, 10mM HEPES buffer, 1mM sodium-pyruvate and 10% FCS and activated using T cell Trans-Act which delivers T cell activation via CD3 and CD28 (Miltenyi) for 2 days in the presence of 100IU/mL rhIL-2 (Aldesleukin). Transact beads were then removed by fully replacing the culture media and T-cells were expanded for 7 days with the addition of fresh medium and 100IU/mL IL-2 every 2–3 days. T-cells were then surface stained with fluorescent antibodies against CD3 (APC-Cy7, Biolegend, CAT# 317341), and CD3^+^ T cells sorted using fluorescent activated cell sorting (FACS). T-cells were then cultured for a further 4 days with addition of fresh medium and 100IU/mL IL-2 every 2–3 days before bulk ICP-MS analysis.

#### Bulk ICP-MS quantification

Activated T-cells were counted and prepared for bulk population ICP -MS analysis on the NexION5000 for the following elements: iron (Fe), magnesium (Mg), and zinc (Zn).

All cell preparation was performed in a highly sterile biological safety cabinet, free from external metals and glass. 4 million cells were placed into a 15mL acid free tube and spun down at 470g for 4 min at 25C. The media was aspirated, and the cells were gently washed with 0.5 mL of HBSS to not introduce bubbles. Centrifugation and washing were repeated for a total of two washes. The cell pellet was fully submerged in 100 *μL* of 65–69% HNO_3_ (Trace Metal Grade) and was left to sit at room temperature for 2 h. The sample was then pipetted up and down, before the volume was pipette measured and recorded. To each sample tube purified water with a resistivity ≥18.2 MΩ cm from an Milli-Q IQ 7015 ultrapure and pure water purification system was added to reach a total sample volume of 300 *μL*. Each sample was subsequently divided evenly in triplicate into acid free tubes and 2.9 mL of type 1 water was supplemented to each sample, with a total volume of 3mL per tube. Samples can be stored for up to two months at 4C.

The samples were run by staff at the London Metallomics Facility and were analyzed by averaging concentrations between technical triplicates and converting the recorded outputs from μg/L to pg/one million cells and fg/single cell.

#### Cell sorting and culture for LA-ICP-MS

PBMCs were obtained as described above. After isolation, cells were surface stained with a panel of fluorescent antibodies which included CD3 (APC-Cy7, Biolegend) CD8 (AF700, Invitrogen) for 30 min at 4°C protected from light. T cells defined by CD3 positive expression using fluorescent activated cell sorting (FACS). After sorting, cells were rested for 40 h in 2mM L-glutamine, 0.1mM non-essential amino acids, 10mM HEPES buffer, 1mM sodium-pyruvate and 10% FCS.

For iron overloading experiments, iron (III) citrate (Sigma) was dissolved in purified water with a resistivity ≥18.2 MΩ cm at a stock concentration of 10mM. Dissolved iron (III) citrate was added directly to cultured cells at a final concentration of 100μM for 10 h prior LA-ICP-MS.

#### Cell preparation for LA-ICP-MS

To adhere the cells to the glass slide, each well of a removable 8-well slide (Labtek) was coated with 150 *μL* poly-L-lysine solution (Sigma) for an hour at room temperature. The solution was aspirated and left to dry for another 30 min with the cover open under sterile conditions free from external metals and glass. 100,000 live cells were counted and placed in MS solution: equal volumes of HEPES (Gibco) supplemented with 2mM EDTA buffer and 4% paraformaldehyde in 1× PBS (Severn Biotech Ltd.) (v/v). The cells were pipetted evenly into each well and allowed to settle at room temperature for 30 min. Each slide was centrifuged at 470g for 4 min at 25°C with brake and acceleration both at 4. Slides were checked under the microscope for attachment and even cell distribution. The supernatant was gently removed via hand pipetting from the sides of the chamber walls ensuring that the tip did not touch the center of the wells. To ensure limited solution contamination, each individual chamber was washed once with purified water with a resistivity ≥18.2 MΩ cm from an Milli-Q IQ 7015 ultrapure and pure water purification system. To limit the “coffee ring” effect, once the chambers were removed, additional manual aspiration was conducted prior to being placed within the laser ablation chamber. Slides were analyzed on the same day.

#### LA-ICP-MS quantification

Quantification of iron in T-cells was performed by Laser ablation Inductively coupled plasma mass spectrometry (LA-ICP-MS). An Iridia 193 nm ArF∗excimer-based LA system (Teledyne Photon Machines), equipped with the cobalt long-pulse ablation cell, was coupled to a Thermo Fisher Scientific iCAP MTX ICP-MS (Thermo Fisher Scientific) via the Aerosol Rapid Introduction System. Human T cell slides were mounted on a four-slide holder and loaded into the cobalt long-pulse ablation.

Tuning of the instrument settings was performed using glass Standard Reference Material (SRM) 612 glass from the National Institute for Standards and Technology (NIST), optimizing for low laser-induced elemental fractionation by monitoring 238U+/232Th+, oxide formation rates (<1%) via the 232Th16O+/232Th+ ratio, as well as the sensitivity of 59Co, 115In and 238U. Counts per second (CPS) for iron were acquired in a fixed dosage mode, with a vertical and horizontal spatial resolution of 20*μm*. The iron isotope of interest was chosen to maximize sensitivity while minimizing isobaric and polyatomic interferences and increasing the signal-to-noise ratio. Dynamic Reaction Collision (DRC) mode with oxygen as the reaction gas was employed in the Collision Reaction Cell (CRC) to reduce the contribution of spectroscopic interferences during imaging. Instrumental drift was corrected using a series of gel standards performed throughout the analysis.

Endogenous element quantification within cells was performed using gelatin micro droplet standards sourced from the Institute of Analytical Chemistry, University of Vienna.^49^ Standard gelatin solutions (fish skin gelatin from Sigma-Aldrich; 10% w/w) were made with increasing amounts of each analyte (0, 173, 351, 684, 1821, 3455 fg) using a multi-element stock solution and single-element standard solutions purchased from LabKings (Hilversum, The Netherlands). Each gelatin standard was fully ablated using the same parameters as the cell samples. The measured intensity of each gelatin pulse response was integrated, and the slope of the calibration curve was employed to convert analyte signal intensities of each cell content measurements.

#### Data analysis for LA-ICP-MS

Data analysis was automated and performed in Python v3.11.5 but can also be done with software provided by Thermo Fisher Scientific. In brief, for each individual chamber and donor, average CPS values for blank measurements were obtained and averaged together. This served as a background threshold for the analysis of Fe (Figure S1A, blue line). Next for each individual cell ablation, any CPS intensity peak that was over the background threshold was used. Peaks on either side of the background were summed together from the first downward peak that was at or below the threshold before and after the CPS intensity peak (Figure S1A, red). This was done individually for each cell input. Combined CPS intensity values were utilized to convert analyte signal intensities of each cell content measurements to fg of iron. The reliability of the regression analysis was verified by assessing the linearity of the calibration curve with correlation coefficients (r^2^) of 0.9986 for iron (Figure S1B).

#### Flow cytometry analysis

T-cells were stained for surface markers CD3 (APC-Cy7, BioLegend), CD8 (Alexa Fluor 700, BioLegend) and CD71 (Percp-Cy5.5, BioLegend), on ice and in the dark for 30 min. Dead cells were excluded from analysis with live/dead staining kit (Invitrogen, L34966). All samples were acquired with a BD LSR Fortessa and analyzed with FlowJo Software (Tri-Star).

### Quantification and statistical analysis

Data analyses were performed with Excel (Microsoft), GraphPad Prism software (v10.3.1), and FloJo (BD Biosciences). Statistical analysis specified in figure legends, and significant results were noted on corresponding graphs with *p* < 0.05. For all data, n corresponds to the number of single cells analyzed with horizontal lines representing the mean values, unless otherwise mentioned in figure legends. For bar graphs in Figures 1H and 2B–2D, the mean ± standard deviation is shown.

## Supplemental information

Document S1. Figure S1 and Table S1

Document S2. Article plus supplemental information
