# Supplementary material for: Single-cell assessment of iron content in primary human T cells using laser ablation inductively coupled plasma mass spectrometry
Source: Cell Rep Methods. 2026 Mar 26;6(4):101343. doi: 10.1016/j.crmeth.2026.101343 (PMC13106974; doi:10.1016/j.crmeth.2026.101343)
Supplement: Document S1. Figure S1 and Table S1 [file mmc1.pdf]

Cell Reports Methods, Volume *6*

Supplemental information

Single-cell assessment of iron content in primary human T cells using laser ablation inductively coupled plasma mass spectrometry

Diana M. Carp, Piotr Golda, Alexander Griffiths, Katie Flaherty, Alexander Morrell, and Anna Schurich

Donor Code	Age (years)	Sex
D1	29	F
D2	28	M
D3	33	M
D4	26	F
D5	27	F
D6	36	M
D7	28	M
D8	50	F
D9	43	M
D10	44	F
D11	62	M
D12	29	F
D13	23	F

Supplementary Table 1: Information on healthy donor characteristics, related to all Figures.

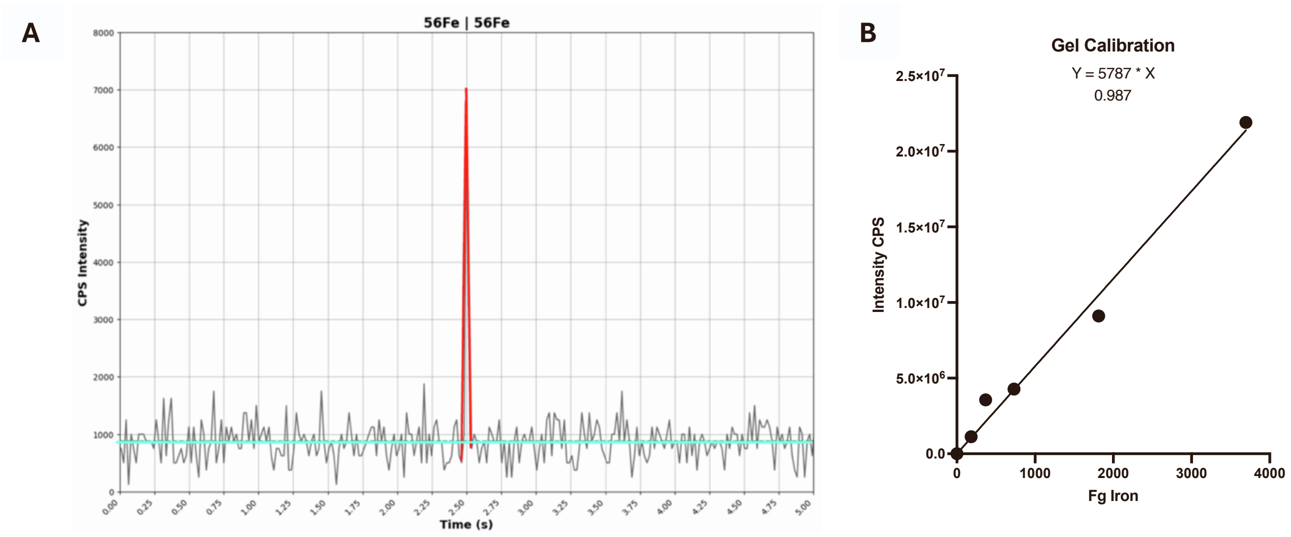

Supplementary Figure 1: Gel calibration for LA-ICP-MS, related to Methods, Data analysis for LA ICP-MS
A) Representative figure of Fe CPS intensity for a single cell (read peak). The blue horizontal line represents the average background threshold when no cell is introduced into the ICP-MS.

B) Regression analysis of ablated gels with known iron content and quantified Fe CPS intensity.
